# Placental cord insertion migration: Implications for ultrasound documentation and follow‐up of abnormal placental cord insertion site

**DOI:** 10.1002/ajum.12399

**Published:** 2024-06-14

**Authors:** Samantha Ward, Zhonghua Sun, Sharon Maresse

**Affiliations:** ^1^ Discipline of Medical Radiation Science, Curtin Medical School Curtin University Perth Western Australia 6845 Australia

**Keywords:** marginal cord insertion, placental cord insertion, placental cord insertion migration, vasa praevia, velamentous cord insertion

## Abstract

**Introduction/Purpose:**

It is well‐documented in the literature that the placenta migrates during pregnancy; however, studies regarding placental cord insertion (PCI) migration are scarce. This longitudinal, prospective study aimed to determine whether PCI migration is a true phenomenon, to assess whether the PCI can change classification during pregnancy and to determine the validity of PCI site documentation including follow‐up of abnormal PCI.

**Methods:**

Eighty‐three participants who had first, second and third trimester ultrasound examinations at a Western Australian private imaging practice over a 12‐month period between November 2021 and November 2022 were recruited. The measured distance of the lower margin of the placenta to the cervix, the distance of the PCI to the closest placental edge and the PCI classification were documented in each trimester. Data analysis was conducted to determine PCI migration rates during pregnancy and to test for association between PCI migration and maternal and placental factors.

**Results:**

The PCI migrated during pregnancy and the PCI classification has the potential to evolve. All identifiable PCIs that were normal in first trimester remained so throughout the pregnancy. The majority (67.6%) of cord insertions that were marginal in first trimester progressed to a normal insertion site by third trimester; 23.5% remained marginal and 8.8% evolved to a velamentous insertion. Three velamentous cord insertions were recorded in first trimester, none of which normalised—two remained velamentous during the pregnancy and one evolved to marginal in second trimester. Marginal cord insertions (MCIs) ≤10 mm from the placental edge in second trimester remained marginal in third trimester; MCIs that were >15 mm from the placental edge in second trimester normalised in third trimester.

**Conclusions:**

Placental cord insertion migration is a phenomenon that occurs during pregnancy with the potential for PCI classification to evolve. Due to the association between abnormal PCI and perinatal complications, coupled with the potential for marginal cord insertion to evolve, documentation of PCI and follow‐up of abnormal PCI is beneficial, particularly in cases of velamentous insertion and marginal insertion at the placental edge or in the lower uterus.

## Background

### Placental cord insertion site

During pregnancy, the placental cord insertion (PCI) site is classified as central, eccentric, marginal or velamentous.^1^, ^2^, ^3^, ^4^, ^5^, ^6^ There are varying definitions of eccentric and marginal cord insertions (MCIs) in the literature. Eccentric cord insertion (ECI) is most commonly defined as a lateral insertion >2 cm from the placental edge^7^ with no documentation in our literature review defining when ECI becomes central PCI. Marginal cord insertion variously characterised as <2.5 cm^8^, <2 cm^2^, ^5^, ^9^ and ≤1 cm^6^, ^10^, ^11^, ^12^ from the placental edge. Occasionally, the cord does not directly enter the placenta; rather, it inserts into the chorioamniotic membranes a varying distance from the placental edge,^8^, ^13^, ^14^ a condition known as velamentous cord insertion (VCI). The vessels of a VCI travel unprotected through the fetal membranes to reach the placenta and are therefore particularly vulnerable. Marginal and VCIs are considered abnormal as they are associated with a variety of obstetric complications,^1^, ^5^, ^8^, ^9^, ^15^, ^16^, ^17^, ^18^, ^19^, ^20^ the most clinically significant being perinatal mortality due to undiagnosed vasa praevia^11^, ^14^, ^21^ whereby unprotected fetal vessels traverse through the fetal membranes within 20 mm of the internal os of the cervix making them susceptible to rupture. There is limited information regarding the incidence of VCI vessel rupture in the absence of vasa praevia although Nagao et al.^22^ describe a case of this nature. While the most significant obstetric complication associated with VCI is vessel rupture due to vasa praevia, there are a number of serious maternal and fetal complications that may occur as a consequence of VCI in the absence of vasa praevia including fetal/perinatal morbidity,^23^, ^24^ placental abruption^8^ and postpartum haemorrhage (>500 mL).^19^ Our literature review suggests this is due to abnormal placental attachment or vascular development.^19^

The current literature confirms the PCI site is readily established on prenatal ultrasound^12^, ^15^, ^21^, ^25^, ^26^, ^27^ with its documentation being widely advocated.^1^, ^11^, ^26^, ^28^, ^29^, ^30^ However, there is considerable variation regarding guidelines for PCI documentation during antenatal ultrasound examinations.^11^, ^31^

### Placental and PCI migration

It is extensively documented in the literature that the relationship between the lower margin of the placenta to the internal os of the cervix evolves during pregnancy^32^, ^33^, ^34^, ^35^ with a high‐resolution rate for low‐lying placenta.^32^ A meta‐analysis by Jansen et al.^36^ describes the resolution of low‐lying placenta as more than 90%. While data regarding placental migration are plentiful, reports of PCI migration are limited. To the best of our knowledge, there are only four studies describing this phenomenon.^10^, ^37^, ^38^ In 1997, Di Salvo et al.^10^ assessed the ability of ultrasound to determine abnormal PCI. They discussed the concept of trophotropism resulting in PCI migration and reported a case of MCI progressing to VCI. Pretorius et al.^37^ investigated the evolution of the PCI in their 1996 study with results suggesting the PCI may change from MCI to VCI and from MCI to normal insertion. More recently, Lutz et al.'s^38^ retrospective study assessed association between the ultrasound‐measured distance of the PCI to the placental margin and perinatal outcomes, demonstrating migration of the PCI during pregnancy, and Wax et al.^12^ described PCI regression from MCI in second trimester to VCI on placental pathological examination.

The purpose of this longitudinal, prospective study was to assess the phenomenon of PCI migration with the aim of establishing evidence‐based information to support, or negate, ultrasound follow‐up of patients with abnormal PCI. We hypothesised that PCI migration is a true phenomenon, there exists a correlation between PCI migration and maternal and placental factors, and the PCI can change classification during pregnancy.

## Materials and methods

### Study design

This longitudinal, prospective study was conducted over a 12‐month period from November 2021 at Vestrum Ultrasound for Women (VUW) clinical research site.

### Recruitment and consent

Patients who attended VUW from 11 weeks' gestation were invited to participate in the study.

Inclusion criteria were as follows:

- pregnant women aged ≥18 years;
- patient is able and willing to give informed consent;
- singleton gestation;
- anterior or posterior placenta; and
- attended VUW for first trimester screening or early anatomy scan, second trimester anatomy scan and a third trimester ultrasound throughout their pregnancy.

A written explanatory statement was provided to each patient (Appendix S1), and written informed consent was obtained (Appendix S2).

### Data collection

A total of 232 patients were recruited for the study, of which 85 patients attended for all three required ultrasound examinations. For each research participant, maternal age and method of conception was recorded. At each obstetric ultrasound visit, the following information was documented:

1. placental location;
2. distance of the lower placental margin from the internal os of the cervix;
3. PCI site:
  - categorised as normal (central and eccentric insertions), marginal or velamentous.
  - distance of the PCI from the closest edge of the placenta
  - closest placental edge (fundal, lower, left, right); and
4. number of cord vessels.

Transabdominal measurements were performed on all patients with transvaginal imaging included if the placenta was low lying or suspected to be low lying. Transvaginal imaging was also included in all cases in which a VCI or MCI was in lower segment to assess for vasa praevia.

For this study, ECI was defined as being between 20 and 25 mm from the closest placental edge (inclusive) and MCI < 20 mm from the placental edge. If the PCI was velamentous, the closest distance from the membranous insertion to the placental edge was measured. This represents the length of aberrant vessels and was documented as a negative value. Central and eccentric locations were collectively considered normal as they are not associated with obstetric complications.^3^, ^25^ For statistical analysis, we stratified the ultrasound examinations into three groups:

- Group 1: First trimester screening/early anatomy scan.
- Group 2: Second trimester anatomy scan.
- Group 3: Third trimester scan.

### Imaging protocol

All imaging was performed by the first author with a second examiner independently documenting measurements of the PCI site to the closest placental edge in first, second and third trimester for 10 randomly selected patients (totalling 30 ultrasound examinations). Ultrasound examinations were performed on a Voluson E10 BT15 ultrasound machine (GE Healthcare Austria GmbH & Co OG) utilising the C1‐5‐D curvilinear 2D transabdominal transducer.

**Figure 1:**
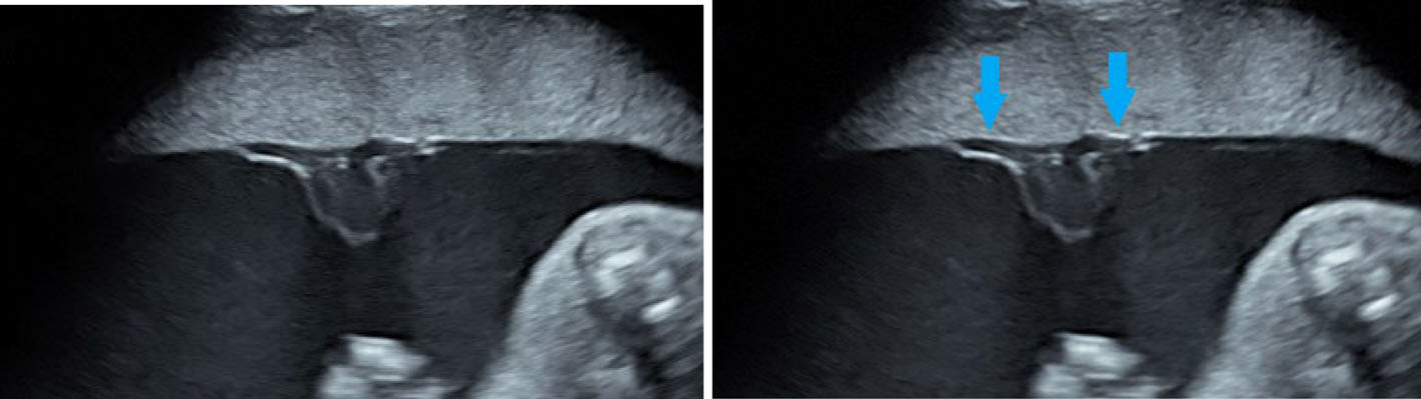
PCI site with vessels branching over the chorionic surface (arrows). PCI, placental cord insertion.

Documentation of the PCI site commenced using B‐mode imaging to identify the point of insertion of the umbilical cord into the placenta. Care was taken to identify the vessels entering the placenta and then branching over the chorionic surface to avoid mistaking a free‐floating loop of cord for the PCI site (Figure 1).

**Figure 2:**
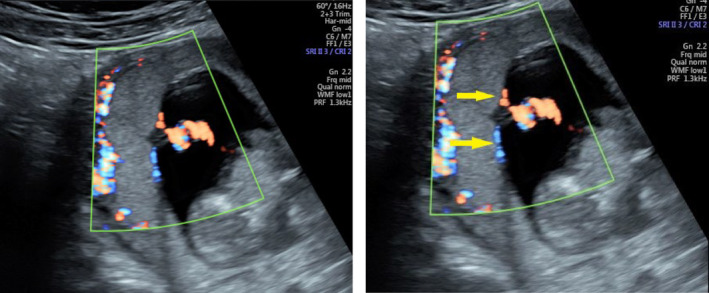
Power Doppler demonstrates the branching of vessels (arrows) at the PCI site. PCI, placental cord insertion.

Colour or power Doppler was applied when necessary to assist locating and confirming the PCI site with demonstration of the vessels traversing the chorionic surface (Figure 2).

**Figure 3:**
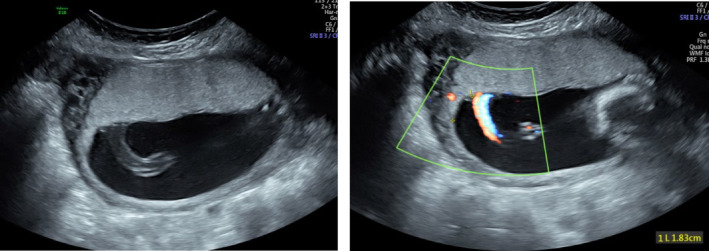
Measurement of the PCI site to the placental edge. PCI, placental cord insertion.

Once the PCI site was established, the ultrasound probe was rotated 360° until the closest distance from the PCI site to the placental edge was identified. It was essential that this method was utilised as imaging the PCI in one plane only can be misleading.^37^ The distance from the PCI to the closest placental edge was measured using a curved distance line, taking care not to include vessels branching over the chorionic surface (Figure 3).

### Data analysis

Data analysis was performed using IBM SPSS Statistics v26 (IBM Corporation, Armonk, NY, USA) and Microsoft Excelv2209 (Microsoft Corporation, Redmond, WA, USA) and G*Power v3.1.9.7 (Heinrich‐Heine‐Universität, Düsseldorf, Germany).^39^ Post hoc analysis using repeated measure ANOVA in G*Power with the effect size of 1.182, level of significance 5% and sample size of 85, calculated the power of our study to be 100%.

Continuous variables were tested for normality using histogram analysis and Kolmogorov–Smirnov testing. All variables were normally distributed except gestational age (P < 0.001). Descriptive data were presented as mean ± standard deviation (SD). Pearson's chi‐squared test was used to compare categorical variables, repeated measures ANOVA determined associations between categorical and continuous variables, and Pearson's correlation tested for association between continuous variables and dichotomous categorical variables. A P value <0.05 was considered to be statistically significant. Intraclass correlation coefficient (ICC) assessed interobserver reliability of the measured distance of the PCI to the closest placental edge. A summary of the statistical analysis can be found in Appendix S1.

### Ethics approval

The study was approved by the Curtin University Human Research Ethics Committee (HRE2021‐0629) and Vestrum Ultrasound for Women (VUW).

## Results

Between November 2021 and November 2022, a total of 85 patients were recruited for this study. Interobserver reliability of measurements of the PCI site to the closest placental edge was found to be excellent with an ICC of 0.98. The PCI was successfully located on B‐mode ± colour Doppler in all first and second trimester examinations. Three patients (3.5%) had PCI sites that were not able to be identified in third trimester; two of these had a posterior placenta; and one had an anterior placenta. All three of these patients had a normal PCI in first and second trimesters.

The participants' mean age at the time of their initial ultrasound was 31.6 years ± 4.9 (range 22–43 years). The average gestational age (GA) at the three ultrasound examinations is demonstrated in Table 1.

**Table 1:** Mean GA at the three ultrasound examinations.

Ultrasound examination	Mean GA in weeks (±SD)	Range of GA in weeks
First trimester screening/early anatomy scan	13.03 (0.76)	11–14
Second trimester anatomy scan	19.76 (0.93)	18–22
Third trimester scan	33.88 (2.35)	28–40

GA, gestational age.

Of the 85 patients, 42 (49.4%) had an anterior placenta and 43 (50.6%) had a placenta that was posterior. There was no significant difference between placental location and the PCI site being normal or abnormal in first, second or third trimesters (P = 0.926, 0.399 and 0.08 respectively). Table 2 shows the frequency of PCI site classification stratified by ultrasound examination.

**Table 2:** Frequency of PCI site classification in first, second and third trimesters (n = 85).

	Normal	Marginal	Velamentous	PCI not located
	n (%)	n (%)	n (%)	n (%)
First trimester screening/early anatomy scan	48 (56.5)	34 (40.0)	3 (3.5)	0
Second trimester anatomy scan	67 (78.8)	16 (18.8)	2 (2.3)	0
Third trimester scan	68 (80.0)	9 (10.6)	5 (5.9)	3 (3.5)

PCI, placental cord insertion.

### PCI migration

The placenta migrates with GA with a mean placental migration rate of 5.32 ± 1.93 mm/week. A significant association (P <0.001) was found between advancing gestational age and increasing distance of the PCI from the placental edge with no association between placental migration rate and PCI migration rate (P = 0.183).

Placental cord insertion migration was calculated as the difference in the distance of the PCI to the closest placental edge between each ultrasound examination, divided by the number of weeks between scans, giving migration per mm per week. The mean rate of PCI migration over the three ultrasound examinations was 1.5 ± 1.49 mm/week. There was a significant association (P = 0.031) when assessing migration rates between groups; the mean migration rate was 2.86 ± 2.49 mm/week, and 1.27 ± 1.38 mm/week between Groups 1 and 2 and Groups 2 and 3, respectively. There was no significant association between the overall PCI migration rate and placental location (P = 0.298), nor was there an association between placental location and PCI migration rates between Groups 1 and 2 and Groups 2 and 3 (P = 0.244 and 0.159, respectively). When analysing data according to whether the PCI site is closer to the fundal or the lower placental edge, the overall migration rate means were 1.11 ± 1.35 mm/week and 2.08 ± 1.51 mm/week, respectively (P = 0.896).

There was a significant difference in the frequency of abnormally sited PCI in second and third trimesters when stratified by conception type. In second trimester, 18% of patients who conceived spontaneously had an abnormal PCI compared with 37% who conceived with assisted reproduction technology (ART) (P <0.023); in third trimester, 13% had an abnormal PCI when conception was spontaneous compared with 62% when conception was assisted (P <0.007). There was a moderate negative correlation between ART and PCI migration rate (r = −0.312, P = 0.033) with a lower migration rate associated with pregnancies conceived through ART. Maternal age (categorised as greater or less than the participants' mean age) did not correlate with the PCI migration rate (r = −0.203, P = 0.172). In addition, there was no correlation between PCI migration rate when stratified by the number of umbilical cord vessels (r = −0.147, P = 0.325).

**Figure 4:**
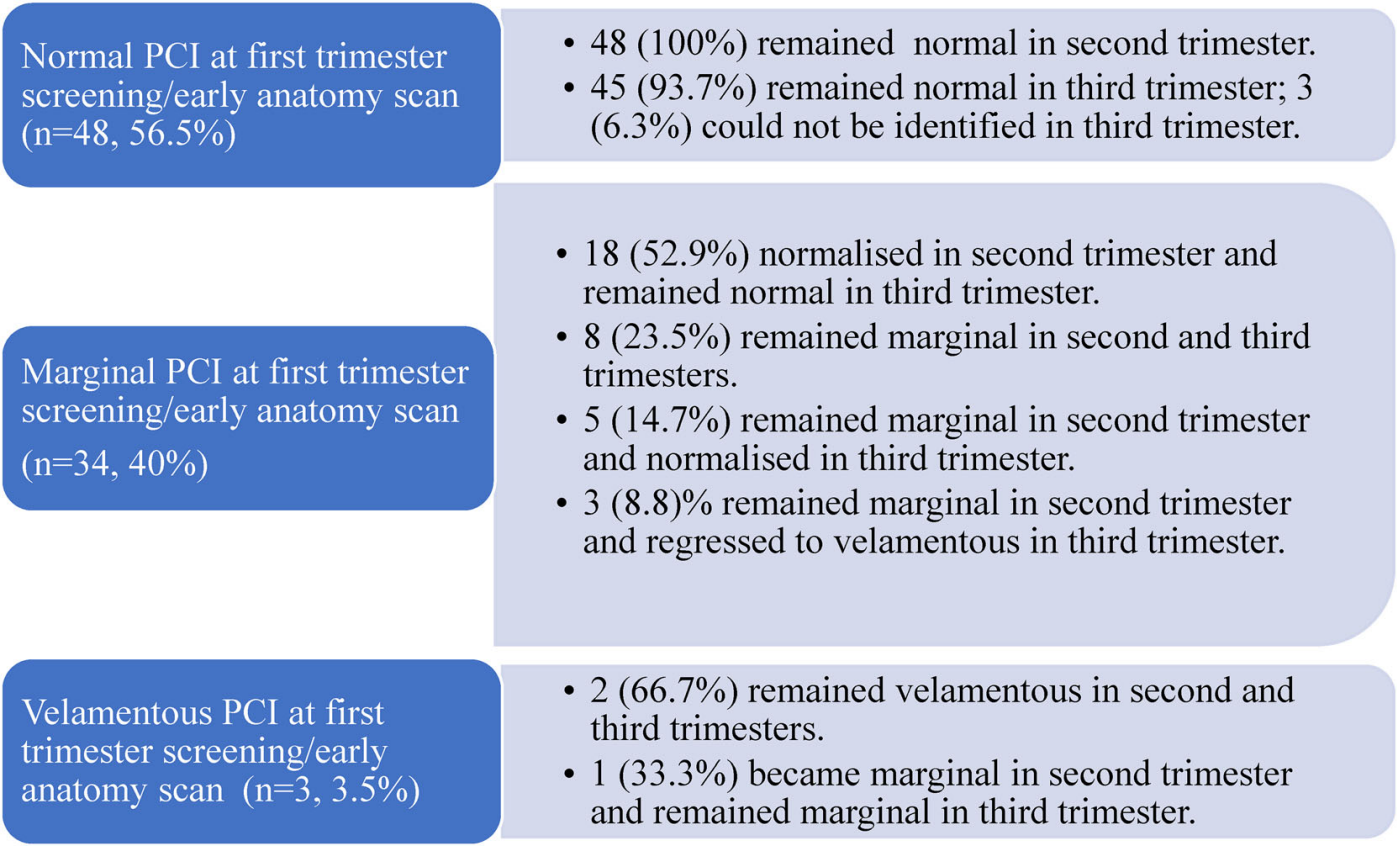
Evolvement of placental cord insertion classification during pregnancy. PCI, placental cord insertion.

Data analysis indicates that not only does the PCI migrate but also its classification can change during pregnancy as shown in Figure 4.

**Figure 5:**
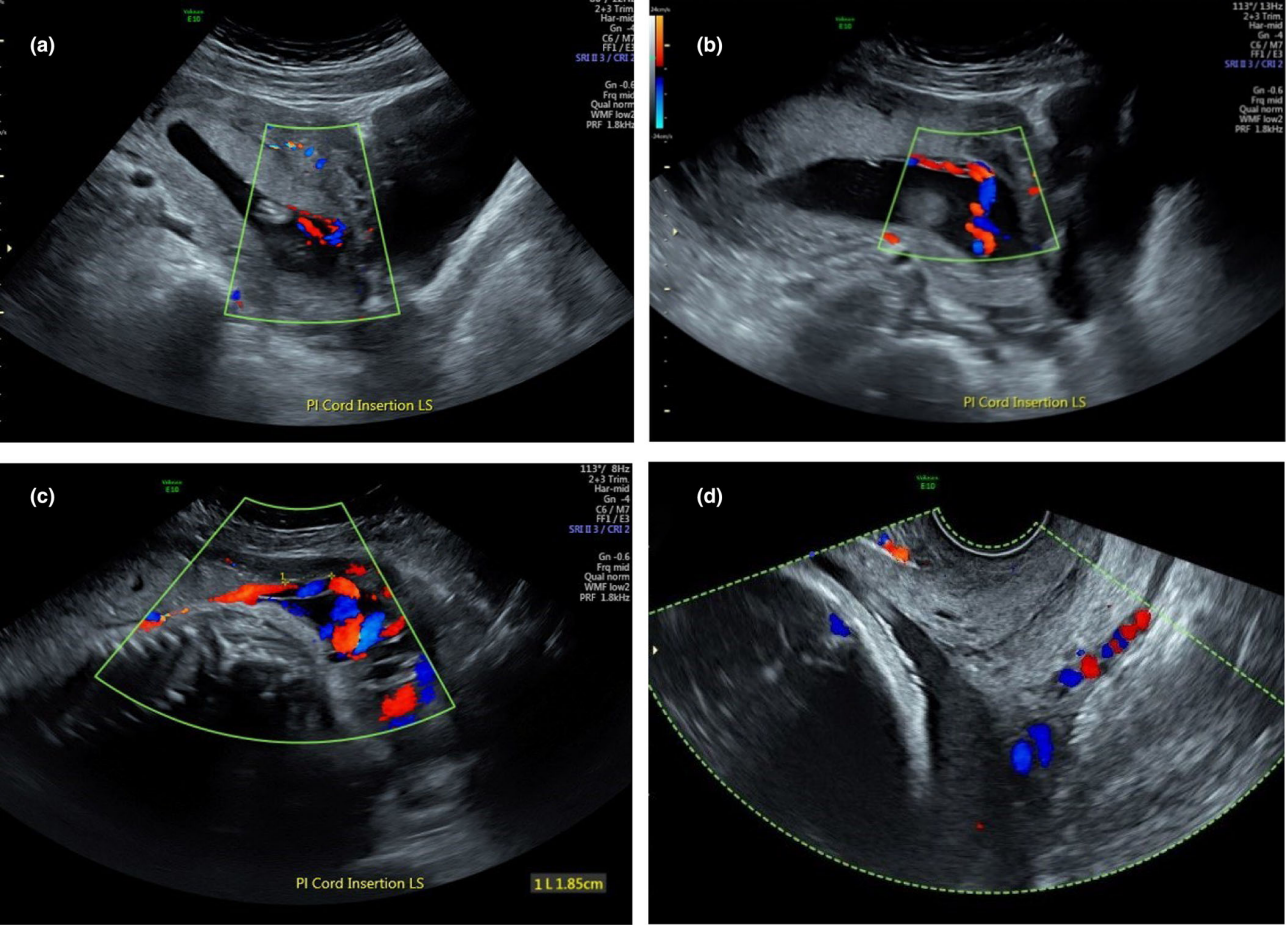
Regression of MCI to VCI. (a) MCI at 13 weeks 5 days (b) MCI at 21 weeks 0 days (c) VCI with 18 mm aberrant length of vessels at 33 weeks 5 days (d) transvaginal image at 33 weeks 5 days demonstrating no associated vasa praevia. MCI, marginal cord insertion; VCI, velamentous cord insertion.

Our results demonstrated that all identifiable PCIs that were normal in the first trimester remained so throughout the pregnancy. The majority (67.6%) of the first trimester MCIs progressed to a normal insertion site by the third trimester with 23.5% remaining marginal and 8.8% evolving to become velamentous. None of the three first trimester VCIs normalised with two remaining velamentous during pregnancy and the other evolving to MCI in second trimester. Of the eight first trimester MCIs that remained marginal in the second and third trimesters, the average distance of the PCI from the placental edge was 10 mm (SD 5.89, range 0–18 mm) in the second trimester. Of the five MCIs that normalised in the third trimester, the mean distance of the MCI to closest placental edge in the second trimester was 15.8 mm (SD 2.59, range 13–18 mm). Of those eight MCIs that remained marginal in all trimesters, seven migrated away from the placental edge by an average of 8.4 mm (SD 4.9, range 1–17 mm) and one migrated 10 mm towards the placental edge. All 3 second trimester MCIs that became velamentous in third trimester were on the placental edge in the first and second trimesters (two at the lower placental edge, one at the fundal edge). The two VCIs in the lower uterus had aberrant lengths of 2 mm and 18 mm; the VCI at the uterine fundus had an aberrant length of 18 mm. Figure 5 demonstrates the regression of the PCI for one of these patients. There was no association between normalisation or regression of a MCI after the second trimester and the closest edge being fundal or lower (P = 0.302).

Table 3 summarises the progression of those PCIs that were abnormal in first trimester with specific reference to whether the closest placental edge was fundal or lower.

**Table 3:** Summary of progression of abnormal first trimester PCI location stratified by closest placental edge (n = 37).

PCI location	n	Fundal	Lower
First trimester–second trimester–third trimester		n (%)	n (%)
M‐N‐N	18	10 (55.6)	8 (44.4)
M‐M‐N	5	5 (100.0)	–
M‐M‐M	8	6 (75.0)	2 (25.0)
M‐M‐V	3	1 (33.3)	2 (66.7)
V‐M‐M	1	1 (100.0)	–
V‐V‐V	2	1 (50.0)	1 (50.0)

M, marginal; N, normal; PCI, placental cord insertion; V, velamentous.

Table 4 shows the average time taken to accurately locate and measure the PCI site to the closest placental edge in 50 randomly selected patients in Groups 1 to 3. There was no association between placental location and time taken in first, second and third trimesters (P = 0.206, 0.546 and 0.092, respectively).

**Table 4:** Average time taken to document PCI in first, second and third trimesters classified by placental location (n = 50).

	n (%)	Average time seconds (±SD)	Range
First trimester screening/early anatomy scan		44.7 (10.5)	22–61
Anterior	18 (36)	44.1 (10.9)	22–61
Posterior	32 (64)	45.0 (10.4)	26–61
Second trimester anatomy scan		51.8 (7.7)	33–63
Anterior	26 (52)	51.7 (8.2)	33–63
Posterior	24 (48)	51.9 (7.2)	38–61
Third trimester scan		59.2 (21.1)	33–121
Anterior	22 (44)	61.0 (26.5)	33–121
Posterior	28 (56)	57.8 (15.2)	34–100

PCI, placental cord insertion.

A summary of the statistical analysis results for this study can be found in Appendix S1.

## Discussion

It has long been documented that the placenta migrates during pregnancy with studies published as early as 1972 describing this phenomenon.^40^, ^41^ While the current literature widely acknowledges placental migration, reports of PCI migration are limited. As mentioned previously, our literature review revealed only four studies describing this phenomenon. To the best of our knowledge, this original research is the first prospective study to investigate PCI migration, migration rates during the three trimesters of pregnancy and correlate these with placental and maternal factors. Our results confirmed that placental cord insertion migration does occur during pregnancy and the classification of PCI can evolve.

The PCI site is readily identified on ultrasound^12^, ^15^, ^21^, ^25^, ^26^, ^27^ particularly prior to third trimester. A study by Sepulveda et al.^21^ in 2003 described 100% visualisation of the PCI in first and second trimesters with a further study by Sepulveda in 2006 describing 100% visualisation of the PCI between 11 and 14 weeks.^26^ Allaf et al.^15^ successfully documented the PCI at 18–20 weeks in 100% of women included in their study. Our results concur with these findings with the PCI site identified in all patients in our study in first and second trimesters. The PCI becomes more challenging to identify in third trimester, particularly when the placenta is posterior;^2^ however, documentation is still feasible. Sepulveda et al.'s^21^ study described only 1% non‐visualisation of the PCI after 30/40 gestation. In our study, we were able to identify all but three (3.5%) PCI sites in the third trimester.

A prospective study by Nomiyama et al.^42^ identified 95% of cord insertions at the second trimester anatomy scan within 60 seconds and a study by Sepulveda et al. identified more than 95% of PCIs during ultrasound examinations from 16 to 40 weeks' gestation in less than 1 minute.^21^ Ugurlucan and Yuksel recorded the average time taken to scan the umbilical cord from fetal insertion to placental insertion in second trimester was 41.5 ± 46.7 seconds.^43^ Our results also demonstrated that inclusion of the PCI site in ultrasound examinations does not unduly extend the scan time with successful documentation of the PCI site taking, on average, less than 1 minute in all trimesters of pregnancy.

It is well‐documented that ART is associated with an increased incidence of VCI in singleton pregnancies.^20^, ^44^, ^45^, ^46^ The precise mechanism behind this association is currently unknown;^47^ however, it is thought that disruption of the multiple sequences required for blastocyst implantation during the ART process may be responsible.^47^, ^48^, ^49^ Our results confirmed this association between ART and VCI and in addition found that PCI migration rates are lower in ART pregnancies suggesting an abnormal PCI is less likely to resolve in an assisted pregnancy than in a spontaneous pregnancy.

In our study, when the PCI was normal in the first trimester, and it was visualised in the third trimester, it remained so for the remainder of the pregnancy. We noticed a decrease in MCI frequency from 40% in first trimester to 10.6% in third trimester, suggesting that in most cases, MCI will migrate in a positive direction, away from the placental edge. In addition, our results suggest a MCI >15 mm from the placental edge in second trimester is more likely to resolve than a MCI 10 mm or less from the placental edge. We demonstrated that the PCIs that were marginal in the second trimester and regressed to velamentous in the third trimester were initially on the placental edge. Our findings imply that PCIs on the placental margin in the second trimester are likely to remain marginal or regress to velamentous in the third trimester.

In our study, 23.5% of those PCIs that were marginal in the first trimester remained marginal in the third trimester and thus continued to carry the risks associated with MCI. In addition, and of great clinical importance, three cases of second trimester MCI became velamentous by the third trimester—there was no associated vasa praevia in any of these cases. Since our study did not include investigation of pregnancy outcomes, it is not known whether perinatal complications occurred in these cases. A recent study by Wax et al.^12^ described PCI regression with three cord insertions that appeared to evolve from MCI in the second trimester to VCI on placental pathological examination, one of which had an outcome of fetal death.

Our study has shown that the PCI migrates during pregnancy and its classification can change over the course of a pregnancy. There are several theories to explain the mechanism of placental migration^32^, ^33^, ^35^ the most widely accepted being that the placenta appears to ‘move’ away from the cervix due to growth of the lower uterine segment and/or placental trophotropism. Placental trophotropism is a phenomenon whereby the placenta grows preferentially towards the region of optimal perfusion at the uterine fundus with atrophy of villi in the lower segment.^33^, ^50^, ^51^, ^52^ We postulate the theory of trophotropism could also explain PCI migration. The PCI changes position relative to the placental edge as the placenta itself remodels its location during pregnancy to maximise its blood supply. This can result in the PCI site being located closer or further away from the closest placental edge as the pregnancy progresses. Regardless of the mechanism of PCI migration, we have demonstrated that the PCI site can progress from abnormal to normal and regress from marginal to velamentous. From an imaging point of view, it would be invaluable if the PCI category and the distance from the closest placental edge (e.g., MCI 7 mm from the fundal placental edge) was documented not only during the ultrasound examination but also in the formal ultrasound report. This would assist in locating the PCI during subsequent scans. If the PCI was normal in earlier examinations, we know that it will most likely remain normal. However, if it was previously abnormal, we need to carefully assess for any regression of the PCI site, particularly if the PCI is in the lower uterus where there is the potential risk of associated vasa praevia. The American Institute of Ultrasound in Medicine (AIUM) and the International Society of Ultrasound in Obstetrics and Gynecology (ISUOG) include PCI as a standard component of the second trimester anatomy scan.^53^, ^54^ In addition, the AIUM includes PCI as a required component of ultrasound examinations between 12 weeks 0 days and 13 weeks 6 days of gestation^55^ and in the third trimester ultrasound examination.^53^ ISUOG recommends third trimester transvaginal imaging when risk factors for vasa praevia are present and for reassessment of a diagnosis of vasa praevia earlier in the pregnancy.^56^ There are no recommendations for documentation of PCI site location in the current Australasian Society for Ultrasound in Medicine (ASUM) guidelines for the performance of first, second or third trimester ultrasound.^57^, ^58^, ^59^

Marginal and velamentous cord insertions are associated with obstetric complications with perinatal mortality due to undiagnosed vasa previa being the most clinically significant. Care must be taken to assess for vasa praevia associated with abnormal PCI, particularly when the cord insertion is in the lower uterus. As previously discussed, there are a number of serious maternal and fetal complications that may occur as a consequence of VCI in the absence of vasa praevia^8^, ^19^, ^23^, ^24^ We have demonstrated MCI can regress to VCI, increasing the potential risk of complications that could be managed when diagnosed prenatally; however, documentation of MCI is currently controversial. One school of thought is that reporting MCIs leads to additional growth scans and unduly increases patient anxiety and thus MCI should not be reported. We propose that while serial growth scans may induce patient anxiety and may be an excessive use of resources, a follow‐up ultrasound at around 36 weeks' gestation for patients with abnormal PCI, particularly when the insertion is at the placental edge or in the lower uterus, could potentially reduce perinatal complications associated with VCI and vasa praevia.

## Limitations

There are several limitations to this study. Since perinatal follow‐up was beyond the scope of this research, PCI location in third trimester was not confirmed on pathology specimens and association between abnormal PCI and perinatal complications has not been established. Our study was performed in a single, specialised obstetric centre and as such our results may not be representative of general radiology practices. Due to the relatively small incidence of laterally located placentas, only those patients with an anterior or posterior placenta were included in the study. Our study would benefit from a larger sample size encompassing patients attending specialised and general ultrasound facilities, with inclusion of perinatal and postnatal outcomes. Incorporating all placental locations and developmental variations may also be beneficial.

## Conclusion

This study confirms PCI migration is a phenomenon that occurs during pregnancy with a realistic potential for PCI classification to evolve. Due to the increased association between abnormal PCI and perinatal complications, coupled with the potential for MCI to regress, we propose documentation of PCI in all trimesters and follow‐up of abnormal PCI in late third trimester would be beneficial, particularly in cases of VCI and MCI at the placental edge or in the lower uterus. Further research conducted in specialised and general ultrasound settings with larger recruitment numbers and inclusion of perinatal outcomes would be valuable.

## Authorship statement

**Samantha Ward:** Design and conception; acquisition of data and method; data conditioning and manipulation; interpretation and discussion; final approval. **Sharon Maresse:** Design and conception; interpretation and discussion; final approval. **Zhonghua Sun:** Design and conception; interpretation and discussion; final approval.

## Conflict of interest

The authors declare no conflict of interest.

## Supporting information


**Appendix S1.** Participant information statement.

**Appendix S2.** Consent form.

**Table S1.** Summary of statistical test used for each statistical analysis.
