# Supplementary material for: Placental cord insertion migration: Implications for ultrasound documentation and follow‐up of abnormal placental cord insertion site
Source: Australas J Ultrasound Med. 2024 Jun 14;27(4):200–9. doi: 10.1002/ajum.12399 (PMC11671736; doi:10.1002/ajum.12399)
Supplement: Supplementary file 1 — Appendix S1. Participant information statement. [file AJUM-27-200-s003.docx]

**S1. SUPPLEMENTORY INFORMATION**


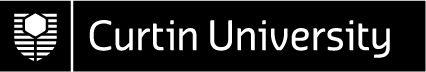

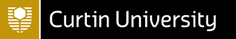


**PARTICIPANT INFORMATION STATEMENT**

**Research Project Title:** Development of a novel ultrasound approach that may reduce the incidence of maternal and fetal complications caused by abnormal placental cord insertion.

**Postgraduate Research Student:** Samantha Ward, Senior Sonographer, Vestrum Ultrasound for Women (VUW).

**HREC Project Number:** HRE2021-0629

**What is the Research Project About?**

During pregnancy, the fetus is connected to the mother via the umbilical cord and the placenta. The site of attachment of the cord to the placenta is known as the placental cord insertion (PCI) and is classified as normal, marginal or velamentous. The umbilical cord most frequently inserts centrally into the placenta (normal cord insertion) but sometimes it inserts towards the placental margin (marginal cord insertion - MCI) or into the fetal membranes prior to reaching the placental body (velamentous cord insertion - VCI). It is well documented in the literature that fetal and maternal complications can be associated with marginal and velamentous cord insertions and the PCI can be readily identified during antenatal ultrasound examinations.

I am a PhD student and my research aims include:

1. Establishing the current practice of PCI site documentation during ultrasound examinations in Australia
2. Evaluating the benefit of further education regarding ultrasound evaluation of the PCI.

Our research to date has demonstrated a wide variation regarding regulation of PCI documentation during antenatal ultrasound in Australia. We anticipate our findings could support a requirement to update protocols for ultrasound documentation of the PCI.

**Who is conducting the Research**?

The research project is being conducted by Samantha Ward, PhD research student, and project supervisors Professor Zhonghua Sun and A/Professor Sharon Maresse.

**Why am I being asked to take part and what will I have to do?**

We would like to hear from you if you have had an adverse pregnancy outcome as a direct result of a MCI or VCI. We understand this is a sensitive issue that can be very difficult to discuss. Our research relies greatly on information and data obtained from women during or after their pregnancy – a time when you may understandably feel particularly anxious or vulnerable. Your personal PCI experience may help us implement a standard PCI site documentation protocol during antenatal ultrasound in Australia, potentially reducing the occurrence of outcomes such as yours.

Your identity as a participant in this research and all information collected will be strictly confidential. Any personal information that could identify you will not be shared or published. You may wish to discuss your involvement in this research project with your partner or other involved parties prior to providing consent and you can withdraw as a participant at any stage.

Your involvement in this research requires you to review and sign our consent form.

**Are there any benefits to being in the research project?**

Although there are no direct benefits to you as a participant of this research project, your experience may contribute to supporting implementation of a standardised ultrasound protocol for PCI documentation. This research could make a significant impact on obstetric outcomes for mothers and their baby/babies affected by MCI and/or VCI.

**Are there any risks, side-effects, discomforts or inconveniences from being in the research**

**project?**

We understand and acknowledge that you may experience anxiety and/or stress by recounting your abnormal PCI cord experience.

We also acknowledge that the time taken to read this statement, ask questions and sign the consent form may be considered an inconvenience.

**Who will have access to my information?**

All information collected will be de-identified and at no stage will it be possible to link your experience to you personally. Only the research team will have access to your information which will be securely stored, and password protected at Curtin University and VUW for a minimum of 7 years after thesis publication.

**Will I be informed of the results of this research?**

We anticipate that the research findings will be made public by means of articles published in peer-reviewed journals, national and potentially international conferences and finally thesis publication. You and the information you provide will be strictly confidential and you will not be identifiable in any of these publications/presentations. As a research participant you can contact the research team (see details below) if you wish to be informed of the results of this research.

**Research team contact details**

Samantha Ward (postgraduate research student): [samantha.j.ward1@postgrad.curtin.edu.au](mailto:samantha.j.ward1@postgrad.curtin.edu.au)

Professor Zhonghua Sun (esearch project supervisor): [z.sun@exchange.curtin.edu.au](mailto:z.sun@exchange.curtin.edu.au)

A/Professor Sharon Maresse (research project co-supervisor): [sharon.maresse@curtin.edu.au](mailto:sharon.maresse@curtin.edu.au)

Thank you for considering participating in this research project.


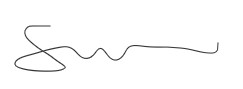


Samantha Ward

Curtin University Human Research Ethics Committee (HREC) has approved this study (HREC numberHRE2021-0629).

Should you wish to discuss the study with someone not directly involved, in particular, any matters concerning the conduct of the study or your rights as a participant, or you wish to make a confidential complaint, you may contact the Ethics Officer on (08) 9266 9223 or the Manager, Research Integrity on (08) 9266 7093 or email hrec@curtin.edu.au.
