# Supplementary material for: Placental cord insertion migration: Implications for ultrasound documentation and follow‐up of abnormal placental cord insertion site
Source: Australas J Ultrasound Med. 2024 Jun 14;27(4):200–9. doi: 10.1002/ajum.12399 (PMC11671736; doi:10.1002/ajum.12399)
Supplement: Supplementary file 2 — Appendix S2. Consent form. [file AJUM-27-200-s001.docx]

**S2. SUPPLEMENTORY INFORMATION**


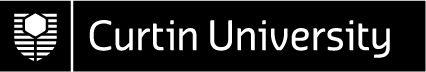

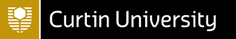


**CONSENT FORM**

| **HREC Project Number:** | HRE2021-0629 |
| --- | --- |
| **Project Title:** | Development of a novel ultrasound approach that may reduce the incidence of maternal and fetal complications caused by abnormal placental cord insertion. |
| **Principal Investigator:** | Professor Zhonghua Sun |
| **Post Graduate Student Researcher:** | Samantha Ward |
| **Version Number:** | 1 |
| **Version Date:** | 25/4/2023 |

- I have read, or had read to me, the information statement version listed above, and I understand its contents.
- I believe I understand the purpose, extent and possible risks of my involvement in this project.
- I have had an opportunity to discuss my involvement in this research project with my partner and/or other involved parties.
- I have had an opportunity to ask questions and I am satisfied with the answers I have received.
- I voluntarily consent to take part in this research project.
- I understand that this project has been approved by Curtin University Human Research Ethics Committee and will be carried out in line with the National Statement on Ethical Conduct in Human Research (2007).
- I understand I will receive a copy of this Participation Information Statement and Consent Form.

| Participant Name |  |
| --- | --- |
| Participant Signature |  |
| Date |  |

Declaration by researcher: I have supplied an Information Letter and Consent Form to the participant who has signed above.

| Researcher Name |  |
| --- | --- |
| Researcher Signature |  |
| Date |  |
