# Supplementary material for: Placental cord insertion migration: Implications for ultrasound documentation and follow‐up of abnormal placental cord insertion site
Source: Australas J Ultrasound Med. 2024 Jun 14;27(4):200–9. doi: 10.1002/ajum.12399 (PMC11671736; doi:10.1002/ajum.12399)
Supplement: Supplementary file 3 — Table S1. Summary of statistical test used for each statistical analysis. [file AJUM-27-200-s002.docx]

S3. SUPPLEMENTARY INFORMATION

**Table 1**: Summary of statistical test used for each statistical analysis.

| Statistical analysis | P value(s) | r value | Statistical test |
| --- | --- | --- | --- |
| Association between placental location and whether the PCI site is normal or abnormal (1st trimester, 2^nd^ trimester, 3^rd^ trimester) | 0.926, 0.399, 0.08 |  | Chi square |
| Association between GA age and PCI distance from closest placental edge | <0.001* |  | Repeated measures ANOVA |
| Association between placental migration rate and PCI migration rate | 0.183 |  | Chi square |
| Association between PCI migration rate between groups 1 and 2 and groups 2 and 3 | 0.031* |  | Chi square |
| Association between overall PCI migration rate and placental location | 0.298 |  | Chi square |
| Association between placental location and PCI migration rates between groups 1 and 2 and groups 2 and 3 | 0.244, 0.159 |  | Repeated measures ANOVA |
| Association between overall PCI migration rate and PCI location (fundal or lower) | 0.896 |  | Repeated measures ANOVA |
| Association between abnormal PCI location and conception type (2^nd^ trimester and 3^rd^ trimester) | <0.023*, <0.007* |  | Chi square |
| Association between PCI migration rate and conception type | 0.033* | -0.312 | Pearson correlation |
| Association between PCI migration rate and maternal age | 0.172 | -0.203 | Pearson correlation |
| Association between PCI migration rate and number of umbilical vessels | 0.325 | -0.147 | Pearson correlation |
| Association between normalisation or regression of MCI after second trimester and the closest edge being fundal or lower | 0.302 |  | Chi square |
| Association between placental location and time taken to document PCI in 1^st^, 2^nd^ and 3^rd^ trimester | 0.206, 0.546, 0.092 |  | Repeated measures ANOVA |

*statistically significant; r, Pearson correlation coefficient
